# A New Successive Time Balancing Time-to-Digital Conversion Method

**DOI:** 10.3390/s23249712

**Published:** 2023-12-08

**Authors:** Konrad Jurasz, Dariusz Kościelnik, Jakub Szyduczyński, Witold Machowski

**Affiliations:** Department of Electronics, AGH University of Science and Technology, 30-059 Krakow, Poland; szyduczy@agh.edu.pl (J.S.); machowsk@agh.edu.pl (W.M.)

**Keywords:** successive approximation, analog-to-digital conversion, asynchronous time-to-digital conversion, self-clocked method, clockless circuit

## Abstract

This paper presents a new self-clocked time-to-digital conversion method based on a binary successive approximation (SA) algorithm. Its novelty consists in combining fully clockless operation with direct conversion of the measured time interval. The lack of any reference clock makes the presented method potentially predisposed to low-power solutions. Furthermore, its circuit representation is extremely simple, thereby the ability to direct conversion of time intervals is not burdened by a significant amount of components. The method is intended to measure relatively long time intervals, i.e., hundreds of microseconds. Therefore, it is suitable for e.g., biomedical applications using time-mode signal processing.

## 1. Introduction

One of the key requirements for modern systems is minimizing energy consumption (low-power electronics). This factor is especially important in systems such as biomedical applications, environmental sensor networks, or commercial mobile devices [1,2,3,4,5]. Regardless of this tendency, since the beginning of the CMOS technology development, the aim has always been to increase the efficiency of the systems by reducing the size of the transistors. For decades, the combination of these two above-mentioned trends has caused the necessity of the gradual supply voltage reduction [6,7].

Digital electronics benefit from the CMOS technological improvements in terms of die area and switching speed [6,7,8,9,10,11]. On the other hand, unfortunately, analog circuit design becomes more and more challenging, because aggressively decreasing voltage headroom and declining transistor threshold voltage deteriorate the signal-to-noise ratio [9,10,11]. This is relevant issue, for instance, for what concerns the dynamic range of analog-to-digital converters (ADCs) [9]. Assuming the same number of bits, a lower power supply means a smaller voltage range per bit.

In reference to this issue, in the last several years, alternative methods of signal processing were proposed, such as time-mode signal processing (TMSP) [6,10,11]. In this technique, instead of processing the information in the conventional, vertical form (voltage domain), the operations are handled horizontally (time domain) using variable time intervals.

**Figure 1:**
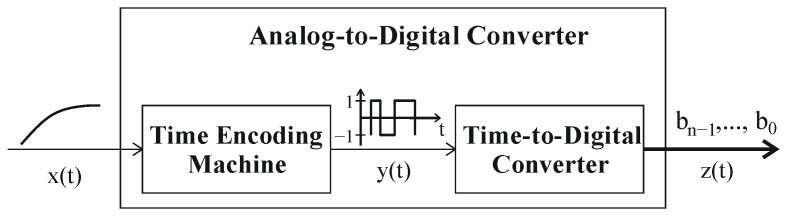
Analog-to-digital conversion implemented using TMSP.

Analog-to-digital conversion using TMSP can be achieved by relatively simple two-stage action (Figure 1). Firstly, by encoding the voltage values x(t) into time intervals y(t), and secondly, by converting them to the appropriate digital words z(t). The migration from the voltage domain to the time domain can be performed, for example, by the time encoding machine (TEM) or the level-crossing sampling technique [12,13,14,15,16]. Such an approach transforms the analog signal to a quasi-digital waveform (voltage quantization, time-length variability) which not only eliminates the shrinking headroom voltage problem, but also provides resistance to undesirable noise and distortion [6,7]. The time intervals generated in this way are then converted to digital words using a time-to-digital converter (TDC).

Depending on the target application, the parameters of both TEMs and TDCs must be carefully chosen to meet specific requirements imposed by the signals that occur in the system. The TEM can be implemented in a few different ways, i.e., as an Asynchronous Sigma-Delta Modulator (ASDM) or as a Spiking Neuron (SN) circuit [17,18,19,20,21,22,23]. A characteristic feature of all TEMs is that they are fully-asynchronous analog circuits designed with an extremely low number of components. It directly translates into advantages such as the low power consumption and the low occupied area, making the TEMs suitable for sensor systems, i.e., pixels of event-based vision cameras [9,24]. However, contrary to the asynchronous operation of TEMs, the conventional TDC solutions require a reference clock which is used for the measurement itself, for the control operations, or both [25,26]. Unfortunately, this is one of the main factors contributing to power consumption [6,12]. For some applications where low-power operation is a crucial design factor, eliminating the clock signal may significantly reduce the energy demand. Therefore, the fully asynchronous time conversion method, which is described in this paper, is suitable for such low-power applications [27,28]. Additionally, it solves the direct conversion problem in the time domain which is described below.

Because of its irreversibility, time does not fit the typical conversion methods commonly used in the voltage domain. A specific moment of an ongoing time interval cannot be directly restored in the same manner as the voltage (current, charge, etc.), which can be decreased, because it is impossible to turn back time. In reference to this issue, preconversion-based time-processing methods are commonly used [10,28,29,30]. They include the prior conversion of the measured time interval to different, decremental physical quantities (i.e., charge), which is then converted to the right digital word. Unfortunately, the preconversion itself is associated, i.a., with the use of additional elements, increased energy consumption, and obviously with additional measurement errors which leads to increased uncertainty of the final result.

The time-to-digital conversion method presented in this paper is based on the binary successive approximation variant which allows converting the time intervals directly, so it is preconversion-free by default [27,28]. In general, there are three binary successive approximation (SA) variants that are adopted in the analog-to-digital conversion: Oscillating Successive Approximation (OSA), Monotonic Successive Approximation (MSA), and Full–Scale Monotonic Successive Approximation (FSMSA) [31]. Each of them has specific properties that are particularly manifested in the issue of direct conversion of time.

**Figure 2:**
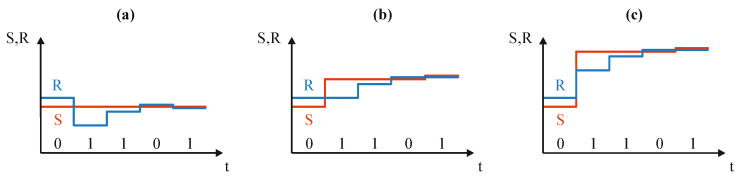
Exemplary waveforms of the successive approximation algorithms: (**a**) Oscillating Successive Approximation (OSA); (**b**) Monotonic Successive Approximation; (**c**) Full-Scale Monotonic Successive Approximation.

The first one—OSA—does not allow for direct time conversion [31]. In this approach, a reference equivalent R of the measured input value S is created (e.g., time interval), based on which the output bits are evaluated (Figure 2a). The equivalent R is created with the use of predetermined binary-scaled reference elements. At each step, the equivalent R is compared to the measured input value S and, based on the result, an appropriate bit is evaluated. If the equivalent is smaller than the measured input value (R < S), the currently tested bit is set to one and the equivalent R is increased by adding the next reference element to it. Otherwise (R > S), the bit is set to zero and the equivalent is decreased by replacing the most recently added reference element with a smaller one. The above operations cause the measured input value to be approximated alternatively, upwards and downwards, causing an oscillatory character (OSA) [31,32]. The necessary reduction of the equivalent R in case of overestimation (R > S) makes the OSA inapplicable as a direct time conversion approach. This is due to the fact that in time conversion the reference elements are time intervals. If the time corresponding to the reference time interval (reference element) has elapsed, it is impossible to return to the moment when the reference time interval started measuring time [31]. This time interval has passed and cannot be turned back in the same way as the voltage or charge can be decreased. Despite this disadvantage, the OSA is by far the most commonly used successive approximation variant and by this, often mistakenly considered to be the only one.

The alternative variant—MSA—has the ability to perform direct conversion of time [31,32]. In this algorithm, the output bits are evaluated based on the successive balancing of the input value S by the binary-scaled reference elements (Figure 2b). In the first step, the measured input value S is compared to the reference R which is equal to the biggest reference element. At each next step, the subsequent reference element is added to this value (S or R) which currently is smaller. In this algorithm, no matter the relationship between the signal S and the reference R values, the compensation is always handled by the addition operation [31]. Thus, the direct time conversion can be successfully implemented using MSA, since the reference elements removal operations (time returning operation) do not occur.

The FSMSA variant also has the ability to produce a direct time conversion, but in comparison to the MSA, it requires twice the number of reference elements—one set for the value R and one for the value S [31,33]. In this case, the digital equivalent of the value S is evaluated on the basis of successive comparisons with monotonically increasing reference value R. The reference value R growth pattern is always the same, regardless of the measured value S. It is created by adding subsequent reference elements at each step of the conversion process. If the value S is smaller than the value R, the overestimation is compensated by adding an identical reference element to the value S that caused the overestimation. After that, the relationship between the values S and R changes and the value S is again greater than the value R [31]. The bit evaluation in this case is similar to the OSA and the MSA. If the value S is smaller than the value R, the bit is set to 0. Otherwise, the bit is set to 1. The FSMSA is rarely applied. One of its few examples of use is [33].

The necessity of using additional reference elements for value S leads to increased energy consumption, making the FSMSA relatively inefficient. This is the second reason (aside from the direct time conversion capability) why the time conversion method presented in this paper is based on the MSA variant rather than the others. It has been named Successive Time Balancing Time-to-Digital Conversion (STB-TDC).

In this paper, the STB-TDC technique is described, with a particular emphasis on time irreversibility. Firstly, a simplified, intuitive model is introduced. It shows how the proposed time conversion method handles the problem of inevitable timelapse. The inherent property of time is its natural, continuous lapse. Neither reversal, stoppage, nor prediction of the measured time interval length is possible. Thus, in direct time conversion, the adopted method has to provide information about the current length of the measured time interval at every moment during the conversion. Only then is the method capable of making correct decisions without any intervening delays which are a source of the additional error. Considering this fact, the continuous timelapse phenomenon must be accurately reflected during the direct time conversion process. The STB-TDC method is based on the well-known SA variant (Monotonic Successive Approximation). Nevertheless, the way it handles the continuous lapse of time is unique and by this it is necessary to describe [27,28]. After this introduction, the circuit representation of the STB-TDC is described along with the processing method relating to the specific elements. Next, the simulation results of the ideal STB-TDC circuit are presented. Finally, the physical implementation of the Successive Time Balancing time-to-digital converter using UMC 0.18 µm technology is presented. The ideal model and the layout implementation are designed using Cadence Virtuoso EDA with the preliminary assumptions imposed in advance by the target technology.

## 2. Idea of the Successive Time Balancing Method

The STB-TDC conversion scheme, as well as the bits evaluation, can be accurately illustrated as a building process of two columns: the signal column *S* and the reference column *R*.

The building components are limited to a single set of *n* binary-scaled, empty reference tanks C_n−1_, …, C_0_ (in real circuits these are capacitors). The capacities of the reference tanks are defined as *C_k_ =* 2*^k^C*_0_, for *k* = 0, …, *n* − 1. In any other consideration, the reference tanks are identical.

The columns S and R are constructed by appropriately stacking the reference tanks C_n−1_, …, C_0_, one on top of another. For every column-building process, the reference tanks are used in the same descending order, starting from the biggest one, C_n−1_.

**Figure 3:**
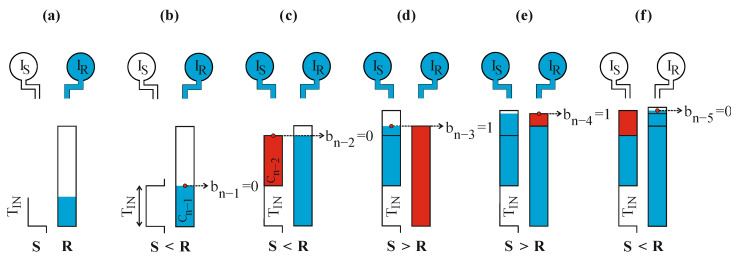
STB-TDC method conversion steps: (**a**) the initial step of the column R building; (**b**,**c**) the initial step of the column S building; (**c**,**d**) adding subsequent tank to the column S; (**d**,**e**) adding subsequent tank to the column R; (**f**) finished conversion process.

Each column has assigned a pump (current source) of constant and equal throughput: I_S_ and I_R_, for the signal column S and the reference column R, respectively (Figure 3). The pumps I_S_ and I_R_ are used to uniformly (constant throughput) fill the subsequent reference tanks with liquid (charge), one after another. Thus, the time required to fill the *k*-th tank C_k_ is always proportional to its capacity *C_k_*.

The conversion process begins with the occurrence of the front edge of the measured pulse T_IN_. At the same moment, the building process of the reference column R begins (Figure 3a). Firstly, the biggest tank C_n−1_ is placed on the column R. Simultaneously, the pump I_R_ is turned on to start filling the biggest tank C_n−1_ with a constant flow rate. The rising liquid level reflects the timelapse of the constantly increasing time interval T_IN_.

The building process of the signal column S starts when the rear edge of the time interval T_IN_ appears (Figure 3b,c). Similarly to the building process of the column R, at that moment the biggest reference tank C_k_ from the empty ones is used (in this example C_n−2_). However, it is placed at the height equal to the length of the time interval T_IN_. Instantly after that, the signal pump I_S_ starts filling the newly connected reference tank C_k_ with a constant flow rate.

All next steps of the conversion process are designated by the moments in time when the filling process of any reference tank is finished. In these moments, two crucial conversion operations are made. Firstly, the appropriate bit is evaluated (corresponding to the specific tank), and secondly, the decision about adding the subsequent, still unused reference tank is made. Following the above, evaluation of the bits b_n−1_, …, b_0_ can be described as follows:

- If the tank C_k_ has been attached to the column S and during its filling process, the filling of a new tank on the (opposite) column R was started, the bit b_k_ is set to logic one: b_k_ = 1 (Figure 3d).
- If the tank C_k_ has been attached to the column R and during its filling process, the filling of a new tank on the (opposite) column *S* was not started, the bit b_k_ is set to logic one: b_k_ = 1 (Figure 3e).
- In any other case, the bit b_k_ is set to logic zero: b_k_ = 0 (Figure 3b,c,f).

The above operations are repeated until the moment when, after filling the tank C_k_, there are no empty ones left that could be used to extend the column (S or R) height (Figure 3f). In the case of the reference tank C_0,_ the bit b_0_ is evaluated as if there were another reference tank, C_−1,_ which would start filling at the moment when the filling of the reference tank C_0_ is finished (Figure 3f).

By looking at the conversion process results statically (omitting the filling), it can be noted that the T_IN_ can be treated as a tank of an initially unknown height. From that perspective, it is successively approximated with the use of preliminary defined reference tanks C_n−1_, …, C_0_ in a similar way as an unknown voltage is determined with a set of voltage references in conventional successive approximation ADCs. In addition, the heights of the columns S and R are equal with the accuracy of C_0_. Because of that, the measured time interval T_IN_ can be evaluated as the difference of the reference elements accumulated on each column.

What is also clearly visible here is the applied variant of the binary successive approximation method (Monotonic Successive Approximation) and its properties [31]. During the conversion process, there is no situation in which it would be necessary to remove any reference tank C_k_ (i.e., subtract reference time interval). This property fits the direct time conversion very well because turning back on time is obviously impossible.

## 3. The STB Time-to-Digital Converter Circuit

Described in the previous section, the column-building model illustrates how the STB-TDC method handles the phenomenon of an inherent and continuous timelapse. However, the model does not present the second greatest advantage of the STB-TDC which is fully asynchronous, self-clocked conversion. In order to show how the particular subcircuits cooperate with each other without any reference clock, it is necessary to migrate from the simplified model to the circuit implementation.

### 3.1. General Architecture of the STB-TDC

**Figure 4:**
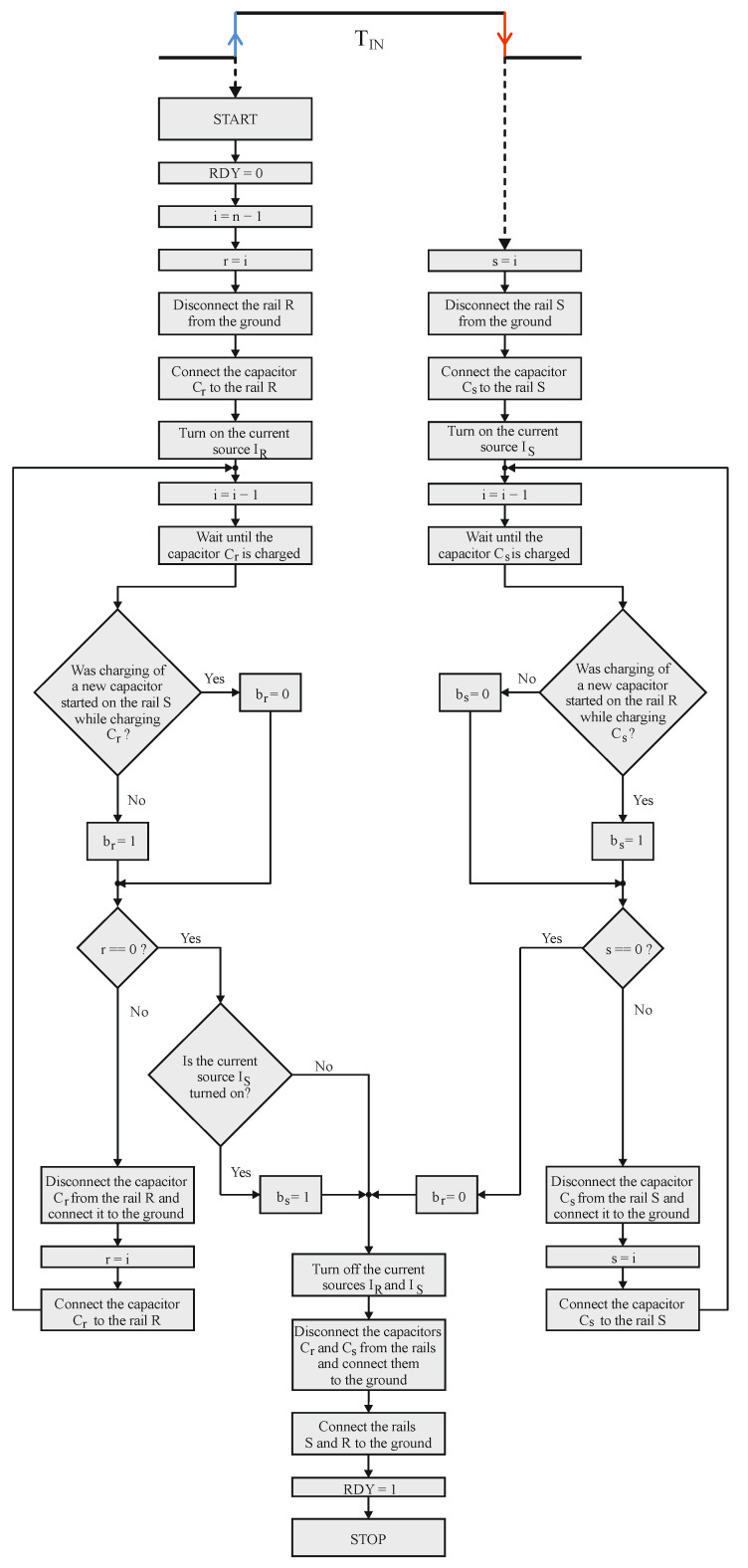
The flow diagram of the Asynchronous State Machine.

**Figure 5:**
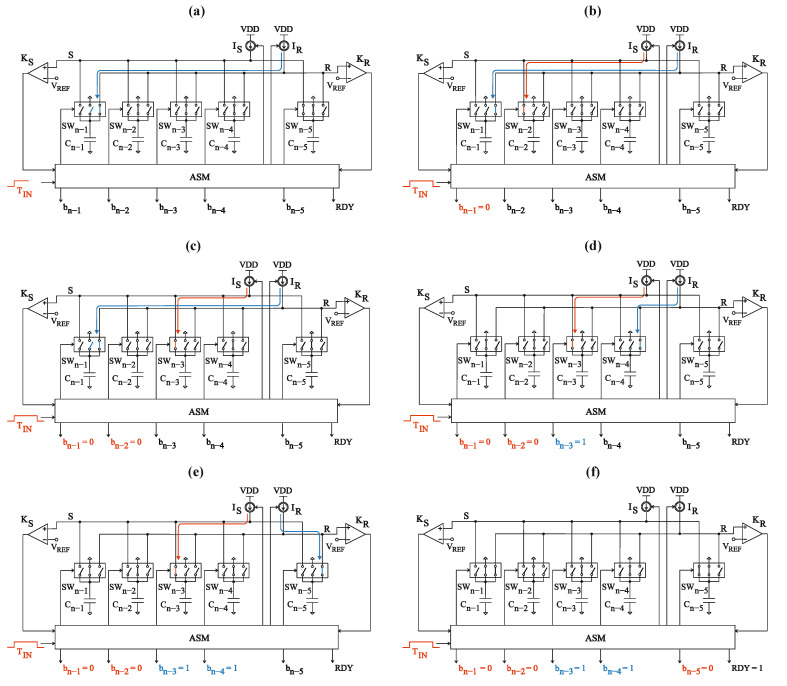
Exemplary states during the STB conversion: (**a**) detection of the rising edge of the measured time interval; (**b**) detection of the falling edge of the measured time interval; (**c**) simultaneous disconnecting the capacitor C_n−2_ from the rail S and connecting the capacitor C_n−3_ to this rail; (**d**) simultaneous disconnecting the capacitor C_n−1_ from the rail R and connecting the capacitor C_n-4_ to this rail; (**e**) simultaneous disconnecting the capacitor C_n-4_ from the rail R and connecting the capacitor C_n-5_ to this rail; (**f**) conversion process finished.

The conversion flow diagram and the complete schematic diagram of the STB time-to-digital converter are presented in Figure 4 and Figure 5, respectively. The fundamental building blocks are: one set of binary-scaled capacitors C_n−1_, …, C_0_, two current sources I_R_, I_S_, two comparators K_R_, K_S_, a reference voltage source V_REF_, a set of analog switches units SW_n−1_, …, SW_0_ and an asynchronous state machine ASM, which controls the operation of the entire converter (Figure 5).

In comparison to the column-building model, there are two rails: S and R, which directly correspond to the signal column S and the reference column R (Figure 3). The reference tanks have been replaced by the binary-scaled capacitors C_n−1_, …, C_0_ defined as *C_k_ =* 2*^k^C*_0_, for *k* = 0, 1, …, *n* − 1.

Each capacitor C_k_ is related to a corresponding bit b_k_, which is evaluated during the conversion process. The capacitors are sequentially connected, one by one, to the rails (S or R) in such a way that one is replaced by another. Thus, at any step of the conversion process, no more than one capacitor is connected to a given rail. Also, with the connection of the capacitor C_k_ to the specific rail, a simultaneous disconnection of the previously connected one is made.

Each capacitor C_k_ has a set of switches SW_k_ associated with it. This set comprises three single switches which allow setting the capacitor C_k_ in one of the three configurations: connected to the rail S, connected to the rail R, or connected to the ground. Initially, all capacitors are connected to the ground and at an appropriate step of the conversion the ASM makes the decision when and where the specific capacitor C_k_ should be connected.

The pumps I_R_ and I_S_ have been replaced by the current sources: I_R_ and I_S_ which are digitally switched on and off. They provide constant current flow to the specific capacitors which are currently used. The charging capacitor with a constant current flow was presented in the column model as filling an appropriate reference tank (Figure 3).

The comparators K_R_ and K_S_ are assigned to the rails in the same way as the current sources I_R_ and I_S_. The purpose of the comparators is to sense the moment when the voltage across a currently charging capacitor reaches the level set by the reference voltage V_REF_. When such an event occurs, the comparator indicates it to the asynchronous state machine ASM which then firstly determines the appropriate bit value, and secondly makes the decision which capacitor should be connected to the specific rail (S or R). Of course, the target rail is always the one on which the charging has just finished.

The asynchronous state machine ASM is a fully clockless control circuit. Neither external (global) nor internal (local) clock signal is required for its proper operation. The only signals it is driven by are the edges of the measured time interval T_IN_ and the event signals generated by the comparators K_R_, K_S_, so basically, the ASM is an event-driven circuit [34]. This is actually where the self-clocked property of the STB comes from. The devices that are directly under the control of the ASM are the switches SW_n−1_, …, SW_0_, and the current sources I_R_ and I_S_. During the conversion process, the ASM connects subsequent capacitors, one by one, to the rail (S or R) via the appropriate switch SW_k_ which is assigned to the chosen capacitor. In addition, it turns on the current source: I_R_ with the T_IN_ front edge and I_S_ with the rear edge occurrence. The conversion process flow diagram of the ASM is presented in Figure 4.

### 3.2. Conversion Process

During the relaxation state, while waiting for the time interval T_IN_, both R and S rails are grounded, which prevents the accumulation of random charges that could disturb the conversion accuracy. For this purpose, the switches SW_n−1_ and SW_0_ are used, respectively, for the reference rail R and the signal rail S.

**Figure 6:**
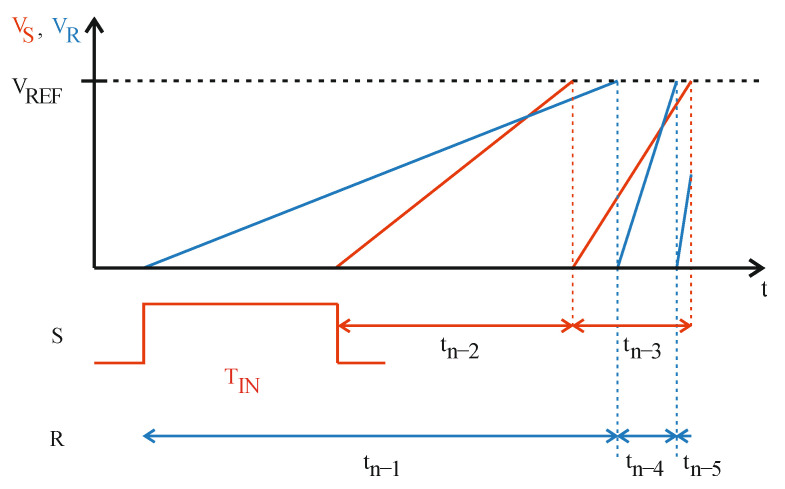
Voltage waveforms of the rails R (blue color) and S (red color).

The conversion starts with the front edge of the measured time interval T_IN_ (Figure 5a). At this moment two actions are performed. Firstly, the triggered ASM simultaneously disconnects the reference rail R from the ground and connects the biggest capacitor C_n−1_ to the reference rail R via the switch SW_n−1_. Secondly, the constant current source I_R_ is turned on causing the linear increase of the capacitor C_n−1_ voltage as well as the reference rail R voltage (Figure 6).

With the rear edge of the measured input value T_IN,_ two operations are performed. Firstly, the biggest capacitor C_k_ (*k* < *n* − 1) from the group of the empty ones C_k_, …, C_0_ is connected to the signal rail *S* via the appropriate switch SW_k_. Immediately after that, the constant current source I_S_ is turned on, which causes the voltage across the capacitor C_k_ and the signal rail S to increase linearly (Figure 5b).

When the voltage across the charging capacitor reaches reference level V_REF_, the relevant comparator (K_S_ or K_R_) triggers the ASM, which performs two operations. The first of them is evaluating the appropriate bit associated with the capacitor that was the most lately connected to any rail. Of course, it does not necessarily have to be the capacitor that has just finished charging and caused the specific comparator to trigger.

The output digital word evaluation can be described as follows:

- If the capacitor C_k_ is connected to the signal rail S, and during the time it is being charged, the charging of a new capacitor on the (opposite) rail R was started, the bit b_k_ is set to logic one: b_k_ = 1 (Figure 5c,d).
- If the capacitor C_k_ is connected to the reference rail R, and during the time it is being charged, the charging of a new capacitor on the (opposite) rail S was not started, the bit b_k_ is set to logic one: b_k_ = 1 (Figure 5d,e).
- In any other cases, bit b_k_ is set to logic zero: b_k_ = 0 (Figure 5a,b,f).

As mentioned before, the exception to the above rules is bit b_0_, because it is associated with the capacitor C_0_ which is connected to one of the rails as the last in the sequence. Thus, in this case, the bit value is determined as if there were another, capacitor C_−1_ that would be used in the processing (Figure 5f).

The second operation after the capacitor C_k_ reaches the V_REF_ is simultaneously disconnecting the fully charged capacitor C_k_ from the rail, connecting it to the ground, and connecting the subsequent capacitor from the empty ones (supposing that there is at least one left). Obviously, it does not necessarily have to be the subsequent capacitor C_k−1_ as it could have been used on the opposite rail already. If there are no capacitors left, the conversion process is finished. The procedure of connecting, charging, and disconnecting the capacitors is repeated until all of them are used (Figure 5f). The duration of the capacitors switching operation is negligibly short, so the current sources I_R_,I_S_ are not turned off when the fully charged capacitor C_k_ is replaced by a smaller one.

Here the self-clocked mechanism is clearly visible. Once triggered, ASM decides about replacing the charged capacitor with an empty one. This newly connected capacitor will become a source that triggers the ASM in one of the later conversion steps.

When the conversion process is completed, the signal RDY is set to the logic one, indicating that the bits determination process is complete (Figure 5f). Simultaneously, the current sources are turned off and the rails are connected to the ground via the switches. The bits b_n−1_, …, b_0_ in the digital output word are latched until the front edge of the next time interval T_IN_ occurs and triggers the conversion process.

## 4. The STB-TDC Circuit Simulations

The proposed conversion method has been verified in the Cadence^®^ Virtuoso environment (version IC6.1.8) by simulating the circuit application (TDC) on the schematic level. The analog parts were designed using ideal elements (comparators, current sources, analog switches, and capacitors) from the default analogLib library. The functionality of the asynchronous state machine ASM was implemented in Verilog-A which is a subset of Verilog-AMS Hardware Description Language. The use of Verilog-A was necessary to fully control the capacitors’ switching sequence and thus prevent the occurrence of false phenomena associated with the use of ideal elements. Since the whole circuit is relatively simple (Figure 5), it was assumed that, overall, it can be supplied using a 1.8 V power source.

The STB method does not strictly dictate the number of used capacitors. As each capacitor C_k_ refers to one bit b_k_, for the purpose of the simulation eight capacitors C_7_, …, C_0_ were used to achieve 8-bit resolution. At such a simplified level of the STB converter the technological aspects can be totally omitted because all the elements are ideal. Still, already at this stage certain parameters should be selected in such a way that they are achievable in the physical semiconductor implementation. The target technology is UMC 0.18 µm which in combination with the purpose of measuring relatively long time intervals (microseconds) leads to the conclusion that for the good circuit matching and high resistance to technological variations, the analog elements should be relatively large in size [35,36]. Following the above, the smallest capacitor C_0_ was set to 500 fF. The reference voltage V_REF_, to which the capacitors C_7_, …, C_0_ are linearly charged, has been set to 1.2 V, which is around the typical value for the real bandgap voltage reference circuits [37,38,39,40,41,42].

The current sources I_R_, I_S_ were set to provide a constant current flow of 1 μA. Thus, the minimum time interval that can be resolved equals to $t_{0}=\frac{V_{REF}\cdot C_{0}}{I_{R(S)}}=600\;ns$, which directly leads to the full-scale range of 153.6 μs.

**Figure 7:**
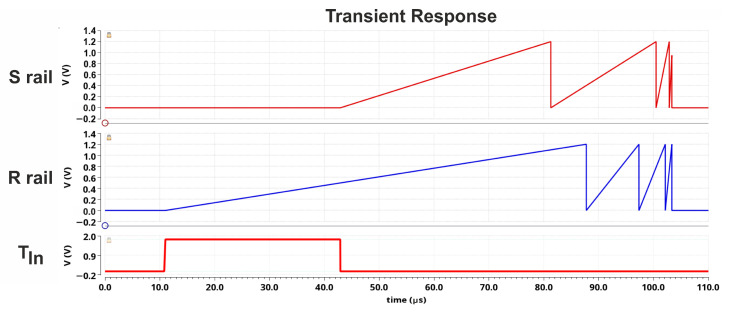
The conversion of input time interval T_IN_ = 31.9 µs.

The verification of the STB converter was carried out using Virtuoso Analog Design Environment XL tool. As the state machine, ASM behavior was handled with Verilog-A, it imposed mixed-signal simulation. Figure 7 presents the result of a single simulation for the measured time interval T_IN_ of 31.9 μs which is converted to the digital word 00110101.

As shown, the STB converter starts the direct time approximation process as soon as the front edge of the measured time interval T_IN_ appears and there is no preconversion process used. The charges generated by the current sources I_R_, I_S_ cause the linear increase of the voltage across subsequent capacitors C_7_, …, C_0_ (one by one). The characteristic, sudden voltage drops indicate the moment of replacing a fully-charged capacitor with another one—not yet used.

**Figure 8:**
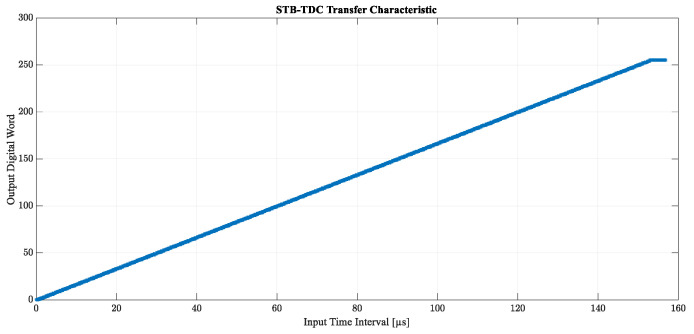
Transfer characteristic of the STB time-to-digital converter based on 3080 measurement points.

The transfer characteristic shown in Figure 8 has been plotted based on 3080 different lengths of T_IN_ in the range from 1 ns to 154 μs with a step of 50 ns. As expected, the results show that at this level of complexity the STB converter is fully error-free and completely coincides with the ideal characteristics of analog-to-digital conversion. This proves that STB-TDC can be successfully used as a direct time-to-digital conversion method and no additional reference clock is needed. The self-clocking mechanism is indeed sufficient to achieve the appropriate quality of conversion.

## 5. The STB-TDC Physical Implementation

**Figure 9:**
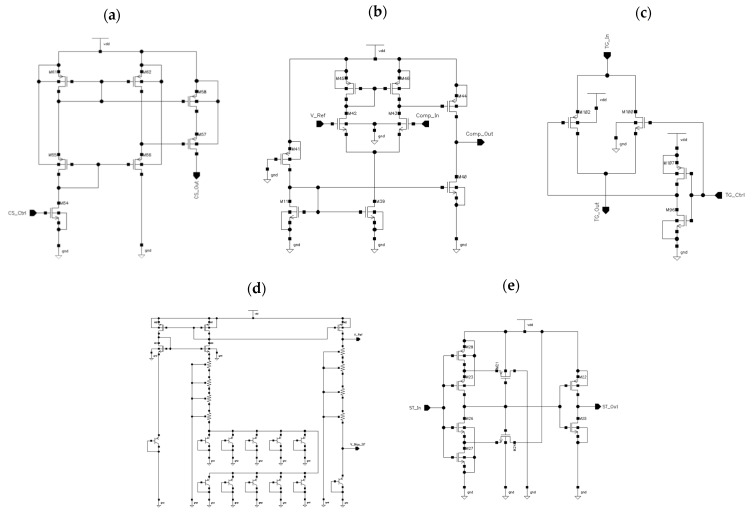
The analog components of the STB-TDC: (**a**) current source I_S_, I_R_; (**b**) comparator K_S_, K_R_; (**c**) one of the three switches comprised in SW_k_; (**d**) bandgap reference voltage V_REF_; (**e**) Schmitt trigger.

The topologies of analog subcircuits used in the implementation of the STB-TDC prototype are presented in Figure 9. The current sources I_S_, I_R,_ shown in Figure 9a, were implemented as the cascode current sources which, by default, provide resistance to the temperature variation, process variation, and channel length modulation effect [43,44,45,46]. In addition, the high-swing modification was applied. It allows the increase of the V_REF_ value to which capacitor C_k_ can be charged without causing the current sources to deviate from the assumed value. For the comparators K_S_, K_R_, shown in Figure 9b, a simple topology has been used which does not require any additional biasing voltage sources [44,47]. Due to the completely clockless design of the STB-TDC, there is no auxiliary reference clock signal. The asynchrony of the comparators K_S_, K_R_ means that their propagation time depends on the rate of voltage change on the monitored rail. Its parameters have been selected in such a way as not to disturb the binary ratio of charging times of subsequent capacitors. Each of the three switches comprised in a single switch SW_k_ has been implemented as a transmission gate (Figure 9c). For 8-bit STB-TDC there are 8 transmission gates (16 transistors) connected to each rail which significantly increases its parasitic capacitance. Therefore, the minimum transistor sizes offered by the UMC 0.18 µm CMOS technology were used for the transistors in transmission gates. The bandgap reference voltage V_REF_ presented in Figure 9d is based on mutual compensation of PTAT-CTAT (proportional to absolute temperature—complementary to absolute temperature) structure [43,44,45,46].

In addition to the above described components, a few circuits have been applied. For instance, a non-inverting Schmitt trigger, shown in Figure 9e, has been connected to the output of each comparator to increase the slope of the edges of the signals entering the ASM [48,49]. In addition, a mutex (mutual exclusion) has been used to avoid the situation of simultaneous appearance of output signals from the comparators K_R_, K_S_ at the input of the ASM. Proper resolution between the signals is absolutely necessary because the circuits related to the individual rails S, R operate independently of each other.

The presented analog circuits can be controlled by a fully asynchronous state machine. As shown, they can be implemented as relatively simple structures. Therefore, they are suitable for direct cooperation with TEM circuits creating time-based ADC conversion system (Figure 1).

**Figure 10:**
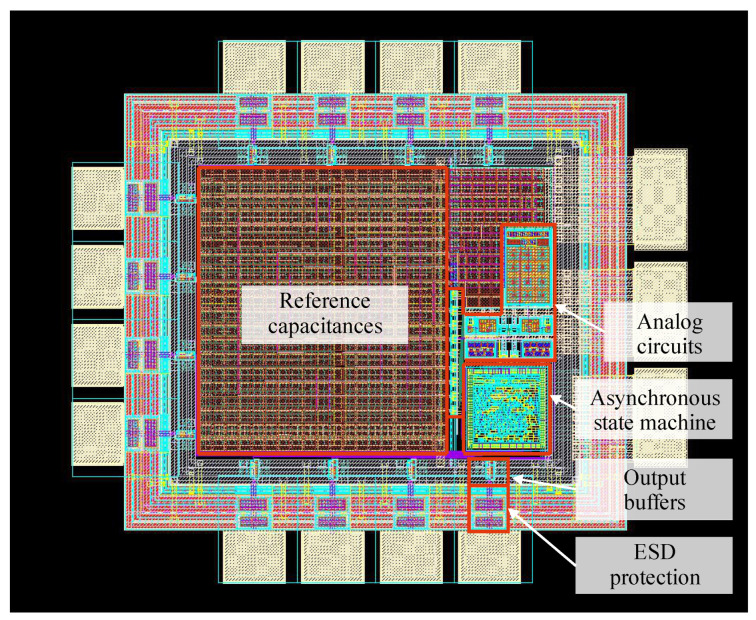
The STB Time-to-Digital Converter layout.

The layout of the first 8-bit STB-TDC prototype is presented in Figure 10. It was designed using UMC 0.18 µm 1P6M CMOS technology. Including the wire bonding pads, the total occupied area equals 1.45 mm^2^ with the aspect ratio of approx. 1.14. The power supply domains for the analog circuits and the digital asynchronous state machine have been physically separated, but both of the domains are dedicated for 1.8 V as it was assumed. The circuit requires two input digital ports: T_IN_, RESET; nine output digital ports: b_7_, …, b_0_, RDY; two power supply ports: VDD, VDDA; and one port for the ground connection: GND which is common for all the components across the chip. Each signal path has been equipped with additional buffers and basic antistatic protection.

The unit capacitor C_0_ has been set to 499.5 fF and the entire reference capacitors matrix was designed using it. The reference capacitors have been arranged based on common centroid algorithm in order to minimize the systematic mismatch. In addition, dummy capacitors have been added around the reference capacitors so that all capacitances have the same neighborhood. Overall, the matrix occupies 0.305 mm^2^ and it is the largest component.

**Figure 11:**
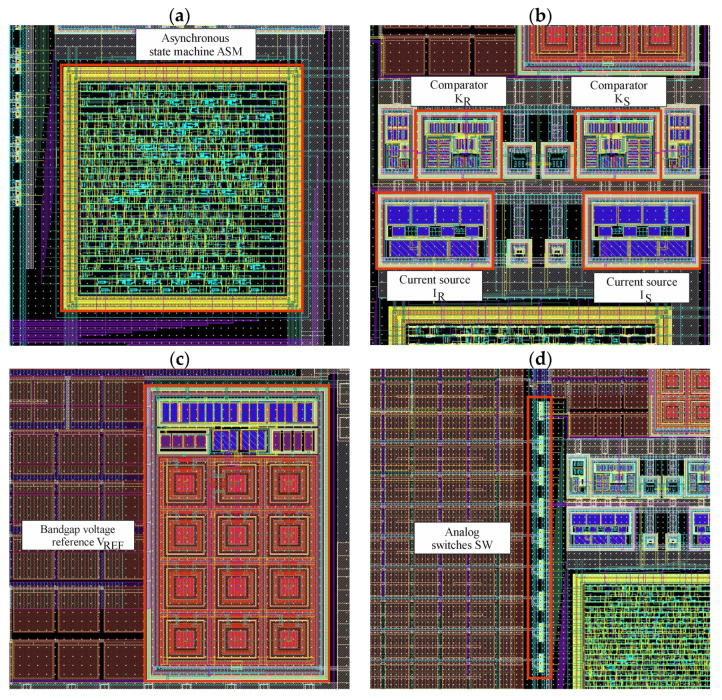
STB-TDC layout subparts: (**a**) asynchronous state machine; (**b**) comparators and current sources; (**c**) bandgap reference voltage; (**d**) analog switches.

The ASM, presented in Figure 11a, has been implemented in Verilog-HDL and synthesized using Genus Synthesis Solution. In the final netlist 359 logic elements from the dedicated library were included to form a fully clockless, event-driven state machine. The physical implementation PnR (place and route) was carried out using the Innovus Implementation System. In order to ensure proper filtration, capacitive filler cells were used. The area of the ASM is 0.03 mm^2^, however, it should be noted that most of it are the above-mentioned filler cells.

The analog circuits are presented in Figure 11b–d. In total, 188 CMOS transistors were used (excluding dummy transistors) to design the whole analog part of the STB-TDC. During the design of each individual components, efforts were made to maintain layout symmetry. The summary area of all the analog circuits is 0.037 mm^2^, most of which is occupied by the bandgap voltage reference as it contains a set of emitter diodes made of PNP transistors.

The post-layout verification of the STB-TDC has been performed in the same way as the verification of the ideal model. Based on the acquired data, selected time-to-digital parameters have been analyzed.

**Figure 12:**
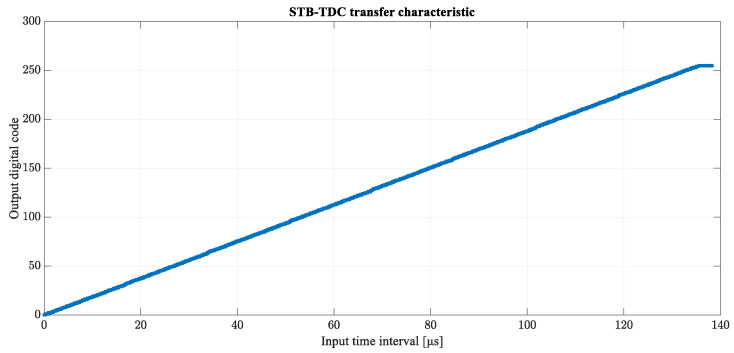
Transfer characteristic of the STB-TDC post-extraction circuit.

Figure 12 presents the final transfer characteristic of the proposed 8-bit STB-TDC design. Comparing the physical implementation to the ideal model, current sources I_R_, I_S_ and bandgap reference voltage V_REF_ values were slightly changed, which resulted in a change of the LSB and therefore in the transfer characteristic. The proposed solution is able to convert the measured time intervals T_IN_ up to 135.66 µs with the LSB of 532 ns. The transfer characteristic is monotone and none of the output codes are missing.

**Figure 13:**
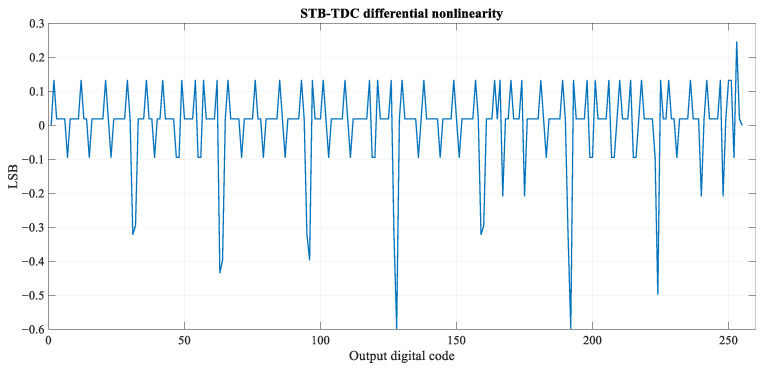
Differential nonlinearity of the STB-TDC post-extraction circuit.

**Figure 14:**
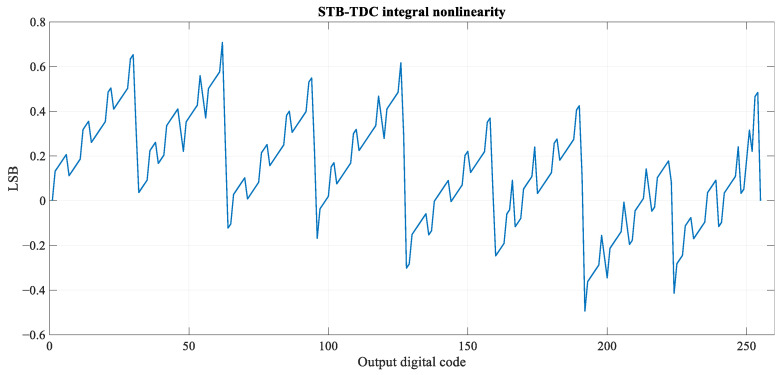
Integral nonlinearity of the STB-TDC post-extraction circuit.

The differential and integral nonlinearity (DNL, INL) plots are shown in Figure 13 and Figure 14. Maximum positive and negative differential nonlinearity errors equal, respectively, 0.25 LSB and −0.6 LSB. The latter occurs for the output digital code transition from 127 to 128. Maximum positive and negative integral nonlinearity equal, respectively, 0.71 LSB and −0.5 LSB.

The average consumed current equals 445 µA, which, after taking into account the power supply of 1.8 V, determines the average power consumption of 801 µW. The above-described parameters are summarized in the Table 1.

## 6. Conclusions

In this paper, a new time-to-digital conversion method based on the binary successive approximation has been presented. Its main advantage consists in a fully clockless operating method with the simultaneous ability to direct conversion of the measured time intervals without the necessity of any preconversion. The introduction of the method was carried out gradually. Firstly, the simplified column-building model has been presented, in which the ability to direct conversion was emphasized. Secondly, the operation of the TDC circuit model has been described, wherein additionally, a fully clockless conversion is clearly visible. Next, the STB-TDC ideal model, followed by the physical implementation using UMC 0.18 µm technology have been presented. Finally, the simulations results as well as the time-to-digital conversion parameters have been shown, which proves that the STB-TDC method can be successfully used for time interval measurement.

## Figures and Tables

**Table 1:** The STB-TDC physical implementation parameters.

Parameter	Value
CMOS process	UMC 0.18 µm
Power supply	1.8 V
Occupied area	1.45 mm^2^
Unit capacitance C0	499.5 fF
LSB	532 ns
Full-scale range	135.66 µs
Maximum DNL	+0.25 LSB, −0.6 LSB
Maximum INL	+0.71 LSB, −0.5 LSB
Average power consumption	801 µW

## Data Availability

The data can be provided upon request from the corresponding authors.
